# The Glycerol Phosphatase Gpp2: A Link to Osmotic Stress, Sulfur Assimilation and Virulence in *Cryptococcus neoformans*

**DOI:** 10.3389/fmicb.2019.02728

**Published:** 2019-11-26

**Authors:** Kevin Felipe Martho, Otávio J. B. Brustolini, Ana Tereza Vasconcelos, Marcelo A. Vallim, Renata C. Pascon

**Affiliations:** ^1^Department of Biological Sciences, Campus Diadema, Universidade Federal de São Paulo, São Paulo, Brazil; ^2^Laboratório Nacional de Computação Científica – LNCC, Labinfo – Laboratório de Bioinformática, Petrópolis, Brazil

**Keywords:** *C. neoformans*, glycerol-3-phosphate phosphatase, *GPP2*, sulfur amino acid biosynthesis, oxidative stress, fungal virulence

## Abstract

*Cryptococcus neoformans* is an opportunist fungal pathogen that causes meningoencephalitis in immunocompromised patients. During infection, this basidiomycete yeast has to adapt to several adverse conditions, especially nutrient availability. The interruption on various amino acid biosynthetic pathways and on amino acid uptake causes reduced viability, inability to cope with various stresses, failure in virulence factors expression and avirulence in animal model of infection. The sulfur amino acid biosynthesis and uptake is an important feature for pathogen survival *in vivo* and *in vitro*. Our previous work demonstrates that *C. neoformans* Cys3 BZip transcription factor controls the gene expression in several steps of the sulfur assimilation and sulfur amino acid biosynthesis. Also, we have shown that Gpp2 phosphatase modulates Cys3 activity. In *Saccharomyces cerevisiae* Gpp2 is induced in response to hyper osmotic or oxidative stress and during diauxic shift. In this work, we will show that, in *C. neoformans*, Gpp2 is required to respond to stresses, mainly osmotic stress; also its transcription is induced during exposure to NaCl. Global transcriptional profile of *gpp2*Δ by RNAseq shows that *CYS3* and other genes in the sulfur assimilation pathway are up regulated, which is consistent with our previous report, in which Gpp2 acts by avoiding Cys3 accumulation and nuclear localization. In addition, several transporters genes, especially amino acid permeases and oxidative stress genes are induced in the *gpp2*Δ strain; on the contrary, genes involved in glucose and tricarboxylic acid metabolism are down regulated. *gpp2*Δ strain fails to express virulence factors, as melanin, phospholipase, urease and has virulence attenuation in *Galleria mellonella*. Our data suggest that Gpp2 is an important factor for general pathogen adaptation to various stresses and also to the host, and perhaps it could be an interesting target for therapeutic use.

## Introduction

Fungal pathogens can cause severe systemic mycoses which are difficult to treat (Garcia-Vidal et al., 2013). One of these pathogens, *Cryptococcus neoformans*, causes fatal meningitis in immunocompromised patients (Powderly, 1993; Limper et al., 2017). This basidiomycete yeast is commonly found in bird excreta and decomposing wood, but it can also colonize the animal host (Kronstad et al., 2012; Ballou and Johnston, 2017; Esher et al., 2018). In order to survive the transition from natural environment to the human host it must adapt quickly to changes, among which, nutrient and osmotic adaptation are essential to survival (Kronstad et al., 2012; Rutherford et al., 2019).

Our previous work showed that nutrient availability is important for virulence and stress resistance. Interruptions on amino acid biosynthesis or failure to uptake amino acids by permeases lead to growth arrest, defects in virulence factor and decreased virulence in mouse and *Galleria mellonella* animal model of infection (Yang et al., 2002; Pascon et al., 2004; Fernandes et al., 2015; Martho et al., 2016; Calvete et al., 2019). Recently, we showed the regulation of sulfur amino acid biosynthesis is essential for *C. neoformans* survival *in vivo* and *in vitro* (de Melo et al., 2019). Cys3 transcription factor, the major regulator of the sulfur uptake network, is part of a protein complex and physically interacts with calcineurin and Gpp2 phosphatase. In *C. neoformans* Cys3 is encountered in the nucleus at high protein levels during sulfur amino acid limitation. The addition of methionine and cysteine to the medium targets Cys3 protein to degradation. Calcineurin regulatory and catalytic subunits are important in order to maintain high levels of Cys3 in the nucleus and Gpp2 seems to be important to send Cys3 to degradation. Deletion of *GPP2* gene causes abnormal accumulation of Cys3 protein (de Melo et al., 2019).

In *Saccharomyces cerevisiae GPP2* is involved in glycerol biosynthesis (Norbeck et al., 1996). In this pathway a glycolytic intermediate, dihydroxyacetone-phosphate is converted into glycerol-3 phosphate by Glycerol-3-phosphate dehydrogenase encoded by *GDP1* and *GDP2* (Albertyn et al., 1994). *GPP1* and *GPP2* encode isoforms of glycerol-3-phosphate phosphatase; these proteins conduct redundant catalytic functions at the last step in this biosynthetic route, the conversion of glycerol-3-phosphate into glycerol (Larsson et al., 1993). *GPP1* and *GPP2* are induced under hyperosmotic shock, partially by the High Osmolarity Glycerol (Hog) pathway. *GPP1*, but not *GPP2*, is controlled by Protein Kinase A activity. The deletion of both genes renders yeast cells sensitive to oxidative stress (paraquat) and especially sensitive to osmotic shock (Pahlman et al., 2001).

Osmoadaptation is also controlled by the calcineurin complex in *S. cerevisiae*. In response to osmotic stress, Ca^2+^ stocks are released from the vacuole to the cytoplasm and functions as a second messenger that leads to dephosphorylation of Ena1 (P-type ATPase on the plasma membrane) facilitating Na and Li efflux, enabling yeast cells to grow on high levels of salt (Cunningham and Fink, 1994; Saxena and Sitaraman, 2016).

Calcineurin has been linked to osmotic stress and cell wall integrity response in *C. neoformans* (Kraus and Heitman, 2003; Fan et al., 2007). Also, the relationship between calcineurin complex and *GPP2* identified in our previous work and others reinforces the idea that this phosphatase complex plays an important role on osmobalance (Park et al., 2016; Jung et al., 2018; de Melo et al., 2019).

In *C. neoformans GPP2* (CNAG_01744) was first identified by a phosphoproteomics approach as a substrate of the calcineurin phosphatase and was later named *HAD1* (Jung et al., 2018). It was hyper phosphorylated (two fold) when calcineurin was inactive (Park et al., 2016). *C. neoformans GPP2* has been considered a target of the mitogen-activated protein kinase Hog1; it is more than two fold induced in wild type in response to osmotic shock and is repressed in a *hog1*Δ strain, suggesting that this gene is a homolog of *S. cerevisiae* glycerol-3-phosphate phosphatase (Ko et al., 2009). Collectively, these data indicate that *GPP2* may be regulated by Hog1 and calcineurin.

In this work, we showed that a *gpp2*Δ strain is highly sensitive to cold shock, membrane, cell wall, alkaline and especially osmotic (NaCl and KCl) stresses. Proline and other amino acids are able to remediate growth arrest caused by osmotic stress. In addition, a global transcriptional profile of the *gpp2*Δ compared to wild type revealed that genes involved in oxidative stress, transmembrane transport and the sulfur amino acid biosynthetic network are induced in the mutant. The association of transcriptomic data and phenotypic analysis of the *gpp2*Δ strain suggests that, there is a connection between oxidative stress, sulfur uptake and osmotic stress response. Whereas, genes related to glucose and tricarboxylic acid metabolism and oxidation-reduction processes were repressed. These data lead us to verify the expression levels of amino acid permease genes, *CYS3* and its target, *SUL1*, by quantitative real-time PCR.

Regarding virulence factors, the *gpp2*Δ strain is unable to produce phospholipase, urease and melanin. *In vivo* virulence assay in *G. mellonella* showed this strain is hypovirulent. In this paper, we propose that glycerol-3 phosphate phosphatase Gpp2 affects sulfur amino acid biosynthetic network, which in turn functions as a central hub which generates metabolites that are essential to counteract osmotic and oxidative stress. In connection with the virulence data this idea underlines the potential of the sulfur network for therapeutic use in *C. neoformans*.

## Materials and Methods

### Medium Composition, Growth Conditions and Materials

Supplementary Table 1 lists the primers used in this work. Growth of *C. neoformans* strains was carried out on YEPD (1% yeast extract, 2% bacto-peptone, 2% glucose); synthetic dextrose (SD) was made with yeast nitrogen base, YNB (0.67 g/L yeast nitrogen base with or w/o amino acid and ammonium sulfate, depending on experimental design, 2% glucose and 10 mM of each amino acid as sole nitrogen source); incubations were carried out at 22, 30 or 37°C on plates or on liquid medium with 150 rpm in a rotary shaker. Amino acids were supplemented at 10 or 20 mM depending on experimental design. Eugenol at 0.1 g/L (Sigma) was incorporated to the YEPD and SD.

### Growth Rate on Amino Acids

Experiments to evaluate the growth on amino acid as sole nitrogen source was carried out on 96 well plates in 100 μL total volume of SD (YNB 1X), 2% dextrose and 10 mM of a single amino acid as sole nitrogen source, according to previous published protocol (Martho et al., 2016). In brief: fifteen amino acids were tested; cells were grown in YEPD at 30°C overnight, collected by centrifugation at 4000 rpm for 5 min and washed three times in sterile 1X PBS (Phosphate Buffered Saline). Intracellular nitrogen pools were exhausted by incubation of the washed cells in 1X PBS at 30°C with 150 rpm rotation for 2 h. After this period 200 cells were inoculated in each well containing a single amino acid as sole nitrogen source. All experiments were done in technical triplicates; plates were incubated at 30 and 37°C for 48 h. The OD_600_ was measured in a plate reader (Epoch 2 microplate reader, BioTek). A minimum of three biological replicates were done for all experiments. The following assay controls were used: inoculums were cultivated on medium with ammonium sulfate as nitrogen source (positive control) and without it (negative control) in the same condition described above. Statistical significance was calculated by one-way ANOVA with GraphPad Prism 7.0.

### Strains and Genetic Manipulation

A reconstituted strain (CNU135) was created by introducing a PCR amplified wild type allele of *GPP2* and a co-transforming plasmid pZPHyg (Idnurm et al., 2004) in CNU125 (*gpp2*Δ:*Nat*^R^) described elsewhere (de Melo et al., 2019) by biolistic transformation (Toffaletti et al., 1993). Confirmation of the genetic modification was done by colony PCR and Southern blot (Supplementary Figure 1). All strains were constructed under the H99 background; therefore, it was used as the wild type control.

### *In vitro* and *in vivo* Virulence Tests

Stress tolerance and virulence factor analysis were done with wild type, *gpp2*Δ:*Nat*^R^ (CNU125 and CNU126) and reconstituted (CNU135) strains. Thermo tolerance was evaluated in rich medium YEPD and SD, with or without ammonium sulfate and amino acids at temperatures of 22, 30, and 37°C. Urea agar was used to determine urease activity (Christensen, 1946). Melanin and phospholipase were assayed according to previously published protocol at 30°C (Paliwal and Randhawa, 1978; Price et al., 1982). Capsule biosynthesis was induced in Sabouraud Broth diluted in MOPS (1:10) at 37°C in shaker (150 rpm) (Zaragoza and Casadevall, 2004). Samples for capsule size evaluation were collected at 24 h and stained with India ink and analyzed by light microscopy (Olympus BX51M). The measurements were performed in biological triplicates with the assistance of CellSens software (Olympus). The data was treated statistically using ANOVA (GraphPad Prism 7).

Multi-stress sensitivity was evaluated with YEPD or SD medium supplemented with 0.5, 0.75, and 1 M of NaCl or KCl. The cell wall and membrane integrity were evaluated on YEPD plus 0.5% Congo Red and YEPD plus 0.03% SDS, respectively. All plates were incubated at 22, 30 or 37°C depending on experimental design.

*In vivo* assays with *G. mellonella* were done according to previous published protocol (Mylonakis et al., 2005). In brief: mutant, wild type and complemented strains were inoculated into 5 mL of YEPD and incubated with orbital agitation 150 rpm for 16–18 h. Subsequently, suspensions were collected by centrifugation, washed twice in sterile 1X PBS and adjusted to 1 × 10^6^ cell/mL in PBS supplemented with ampicillin (20 mg/kg body weight). Groups of 16 caterpillars with 200 mg of average weight were inoculated with 10 μL of the suspension with a Hamilton syringe in the last pro-paw. Thereafter, caterpillars were separated on glass Petri dishes (15 mm diameter) and incubated at 30 and 37°C during 8 days. They were monitored daily by observing spontaneous or induced movements with sterilized tweezers. The experiment was completed when the larvae died or formed cocoons.

### qPCR

Total RNAs were obtained from wild type and mutant strains incubated overnight in liquid YEPD under 150 rpm agitation at 30°C. RNA extraction protocol was described before (Calvete et al., 2019). cDNA synthesis was done with 5 μg of total RNA, with RevertAid H minus First Strand cDNA synthesis kit (Thermo Scientific), Oligo dT and random hexamer primers. Real time PCR amplifications were made from diluted cDNA templates (1:10) with 600 nM target primers, 300 nM GPDH1 (Glyceraldehyde-3-phosphate dehydrogenase) internal control primers, and 1X SYBR Green (EvaGreen^®^). Quantification of the transcript levels was performed in StepOne thermo cycler (Applied Biosystems), normalizing gene was done with GPDH1 according to Livak and Schmittgen (2001). An analysis of variance was performed by Tukey’s multiple comparison test using GraphPad Prism 7.0 software, and *p* values lower than 0.05 were considered statistically significant.

### Transcriptional Profile: RNA Extraction, Quantification, and RNAseq Analysis

All RNAseq experiments were performed in triplicates. Strains (mutant and wild type) were grown overnight in YEPD at 30°C with 150 rpm rotation. After this period, the cells were induced in liquid YEPD for 2 h (30°C) at 150 rpms. Total RNA was extracted as described above. RNA quantification, purity and quality were evaluated in NanoDrop spectrophotometer (Thermo Scientific, Waltham, MA, United States) and RNA Nano 6000 Assay Kit of the Bioanalyzer 2100 system (Agilent Technologies, Santa Clara, CA, United States). Libraries were made from 4 μg of total RNA according to instructions of the Illumina TruSeq Stranded mRNA Sample Prep LS Protocol. Library quantifications were performed by qPCR using the KAPA Illumina qPCR Quantification Kit. Quantification and validation of the libraries were done by quantitative PCR and they were diluted at the working concentration (2 ηM); libraries were denatured with sodium hydroxide solution at 0.1 N and then diluted to 20 (M and loaded into HiSeq sequencer with the v4 sequencing kit (2 × 100 cycles). Sequencing was performed at Centro de Genômica Funcional – ESALQ/USP, Piracicaba, Brazil.

The raw fastq files were filtered by the software BBDuk2^1^ which is capable of quality-related trimming and filtering adapter-contaminant. Any Illumina adapters and reads with low quality (bellow Q30) were removed. The FASTQC^2^ created the overall quality of the sequencing reports. The filtered reads were mapped into the *C. neoformans* H99 genome sequence using the software STAR aligner (Dobin et al., 2013). The genome and annotation files were retrieved from the FungiDB database^3^. The R/Bioconductor package Rsubread/featureCounts (Liao et al., 2019) made the counting table for the posterior statistical analysis. The Differential Gene Expression (DGE) test was performed by the R/Bioconductor package DESeq2 (Love et al., 2014) with the method of shrink log-fold changes presented at the package apeglm (Zhu et al., 2018). The genes with adjusted *p*-value (corrected by false discovery rate – FDR) below 0.05 and log2 fold change above or below 1.0, were considered as differentially expressed (DEG). The Gene Ontology (GO) enrichment analysis was performed by the R/Bioconductor package GOstats (Falcon and Gentleman, 2007). The package Pathview (Luo and Brouwer, 2013) was used to visualize the KEGG pathways. The protein-protein interaction network was created using the STRING database (Szklarczyk et al., 2017).

## Results

### *C. neoformans* CNAG_01744 Encodes a Glycerol-3-Phosphate Phosphatase (*GPP2*)

*Cryptococcus neoformans* CNAG_01744 was identified in our earlier research by immune precipitation as part of a protein complex with the major sulfur amino acid biosynthesis transcription regulator Cys3 and the calcineurin catalytic and regulatory subunits Cna1 and Cnb1, respectively (de Melo et al., 2019). We named this gene as *GPP2* based on the amino acid sequence similarity to *S. cerevisiae GPP2*, a glycerol-3-phosphate phosphatase (Pahlman et al., 2001).

Previously, this gene was also found in *C. neoformans* by phosphoproteomics approach as substrate for the calcineurin complex (Park et al., 2016) and later it was named as *HAD1* (Halo Acid Dehalogenase hydrolase) due to the predicted molecular function (Jung et al., 2018). *C. neoformans* bears four genes that encode Halo Acid Dehalogenase hydrolase-like proteins (CNAG_06132, CNAG_01744, CNAG_6122, and CNAG_06698). BLAST similarity searches and amino acid sequence alignments indicated that CNAG_01744, CNAG_06122, and CNAG_06132 may be homologs of *S. cerevisiae* Gpp1 and Gpp2 (glycerol-3-phosphate phosphatase), which can also be classified as a HAD-like dehalogenase. However, the percentage of identity among these amino acid and *S. cerevisiae GPP2* sequences are very close (29.9, 31.1, and 31.4% identity, respectively), suggesting that sequence similarities may not be the best tool to indicate which one of these genes is a homolog of Gpp2. Alternatively, glycerol-3-phosphate phosphatase (Gpp2) may be encoded by more than one gene, as it is the case in *S. cerevisiae*, in which *GPP1* and *GPP2* encode very similar proteins, that have the same catalytic function, but are differentially regulated according to the environmental condition (Pahlman et al., 2001). In this paper, among other data, we will present phenotypic features of *gpp2*Δ which suggest its role as an important genetic element involved in osmotic stress response. Also, we will refer to CNAG_01744 as *GPP2*.

### *GPP2* Is Involved in Cold Shock and Nutritional Stress

**FIGURE 1:**
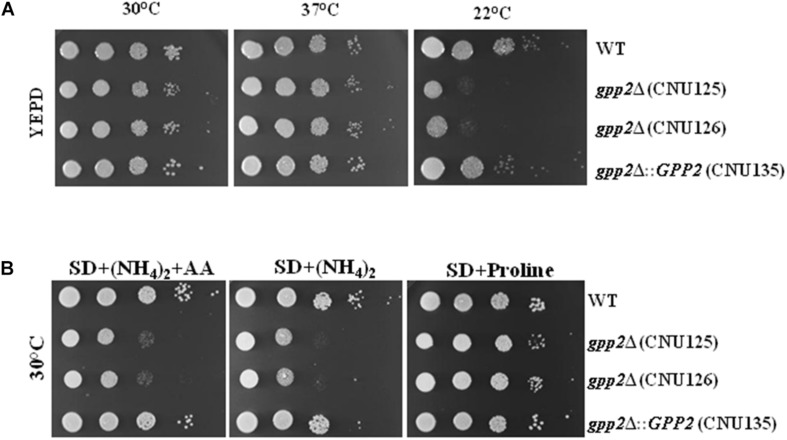
Growth pattern of the *gpp2*Δ mutant (CNU125 and CNU 126) compared to wild type (H99) and reconstituted strain (CNU135) in **(A)** YEPD and three different temperatures (22, 30, and 37°C); **(B)** the same strains on Synthetic dextrose (SD) supplemented with ammonium sulfate and amino acids (tryptophan, histidine, and methionine), SD with ammonium sulfate only and SD supplemented with 10 mM of proline as the sole nitrogen source at 30°C. Spot dilutions are 1 × 10^5^ to 1 cell at the last spot.

We tested several stress phenotypes associated to *gpp2*Δ strain relative to wild type and a reconstituted strain (CNU135) which was constructed in this work (Supplementary Figure 1). As shown in Figure 1A, deletion of *GPP2* did not significantly changed growth in rich medium (YEPD) at 30 and 37°C. However, cold shock (22°C) caused severe growth arrest in *gpp2*Δ strains relative to wild type and reconstituted strains in YEPD (Figure 1A). There was also, growth deficiency in SD supplemented with the preferred nitrogen source (ammonium sulfate) and/or amino acids (Figure 1B), suggesting that, in this condition, the mutant strain may have some sort of nutritional deficiency. Interestingly, growth arrest in SD was reverted in the presence of 10 mM of proline as sole nitrogen source (Figure 1B).

This result led us to investigate if other amino acids could rescue the low growth phenotype in SD. Table 1 shows the growth rate of two mutants compared to wild type in single amino acid as sole nitrogen source at 30 and 37°C in liquid medium. Besides proline, methionine, glutamine, asparagine, and aspartate could complement the growth defect in SD as sole nitrogen source, yielding growth rates that are not statistically different (NS) than wild type in the same amino acid as sole nitrogen source. It is noteworthy to mention that glutamic acid, raised growth rate to more than 70% compared to wild type in spite of a significant statistical difference between mutant and wild type (*p* < 0.001). It is important to mention that all these amino acids, except methionine, support growth in *C. neoformans* wild type to the same rate as the preferred nitrogen source (ammonium sulfate). Leucine as sole nitrogen source also improved the growth rate of the mutants to wild type levels (CNU125 100% and CNU126 97%, NS), but only at 37°C.

**TABLE 1:** The growth percentage of two independent *gpp2*Δ strains (CNU125 and CNU126) in 10 mM of each amino acid relative to wild type at 30°C and 37°C.

**% growth relative to wild type**												

**N^#^**	**30°C**						**37°C**					
		
	**CNU125**	**SD**	***p***	**CNU126**	**SD**	***p***	**CNU125**	**SD**	***p***	**CNU126**	**SD**	***p***
NH_4_	27,6	1,9	^∗∗^	24,0	3,9	^∗∗^	50,6	12,4	^∗^	55,4	10,9	^∗^
GLY	44,8	6,2	^∗∗^	42,3	5,7	^∗∗^	61,0	3,4	^∗^	71,2	6,4	^∗^
LEU	38,2	8,9	^∗∗∗^	36,1	11,2	^∗∗∗^	100,3	5,6	NS	97,8	22,2	NS
ILE	75,4	1,8	^∗^	69,7	4,4	^∗^	54,1	0,8	^∗^	61,7	5,5	^∗^
VAL	20,0	1,4	^∗∗^	21,0	2,9	^∗∗^	42,4	6,9	^∗∗^	43,1	6,4	^∗∗^
SER	25,2	5,1	^∗∗∗^	24,2	5,9	^∗∗∗^	56,0	10,5	^∗^	29,8	1,7	^∗∗^
MET	99,3	7,7	NS	87,7	2,8	NS	87,2	19,4	NS	80,3	3,5	NS
ASP	104,3	11,9	NS	101,6	4,9	NS	67,16	13,62	NS	71,54	7,9	NS
ASN	93,4	14,4	NS	101,6	4,9	NS	107,6	15,7	NS	101,1	2,5	NS
GLU	70,7	2,8	^∗∗^	68,3	2,6	^∗∗^	81,0	0,7	NS	76,9	19,0	NS
GLN	90,0	8,5	NS	91,7	8,7	NS	85,2	7,2	NS	98,5	1,5	NS
ARG	39,3	4,6	^∗∗∗^	34,2	7,3	^∗∗∗^	57,7	7,6	^∗∗^	51,2	6,0	^∗∗∗^
LYS	51,7	0,3	^∗∗^	66,8	4,1	^∗^	89,3	10,0	NS	105,3	0,5	NS
PHE	55,8	13,0	^∗^	54,2	3,9	^∗^	46,6	8,1	^∗^	37,5	9,9	^∗∗^
TRP	15,4	2,8	^∗∗∗^	18,6	2,3	^∗∗∗^	36,7	3,9	^∗∗^	26,2	4,3	^∗∗^
PRO	113,2	2,3	NS	97,2	5,4	NS	119,8	14,1	NS	82,4	0,4	NS

*^#^Nitrogen source. NS means that differences are non-significant between mutant and wild type and the meanings of asterisks are: ^∗^*p* < 0.005, ^∗∗^*p* < 0.001 and ^∗∗∗^*p* < 0.0001.*

### *GPP2* Plays an Important Role in Membrane, Cell Wall and Osmotic Stress Responses

**FIGURE 2:**
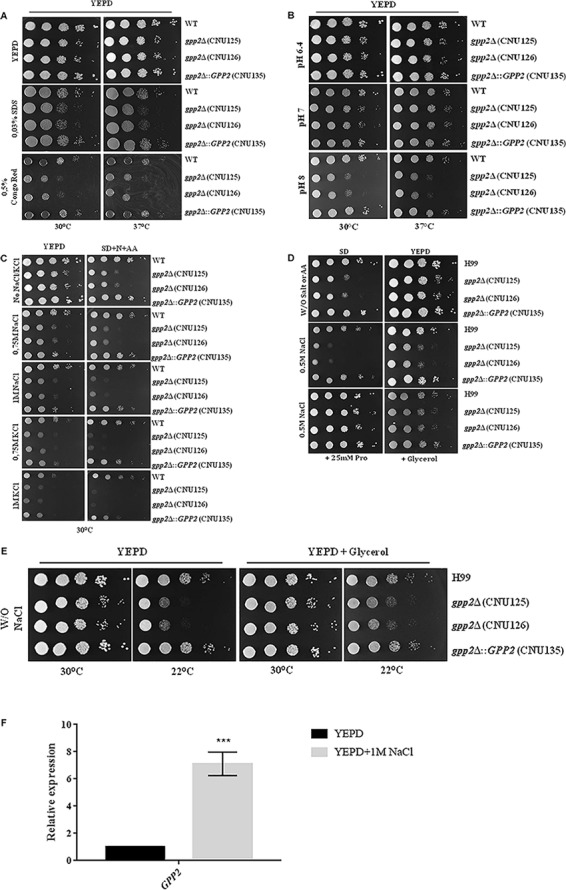
Growth rate of *gpp2*Δ mutant (CNU125 and CNU 126) compared to wild type (H99) and reconstituted strain (CNU135) in **(A)** YEPD, YEPD supplemented with 0.03% of SDS and YEPD supplemented with 0.5% of Congo red. **(B)** Growth rate of the same strains in different pHs (6.4, 7.0, and 8.0) at 30 and 37°C and **(C)** growth rate of the same strains in the presence of osmotic stress (0.75 and 1 M of NaCl and KCl) in YEPD and SD supplemented with the preferred nitrogen source; **(D)** growth rate of gpp2Δ mutants, wild type and reconstituted strain in SD, SD supplemented with 0.5 M NaCl and SD plus 0.5 M NaCl and 20 mM of proline (left panel), the same strains in YEPD, YEPD supplemented with 0 M NaCl and YEPD plus 0.5 M NaCl and glycerol (right panel); **(E)** effect of the temperature (22°C) on wild type, reconstituted and mutant strains in YEPD with and without glycerol. Spot dilutions are 1 × 10^5^ to 1 cell at the last spot. **(F)** qPCR showing the induction of *GPP2* transcript in the presence of 1 M NaCl relative to the control YEPD in wild type strain H99. Statistical significant differences were tested by Two-way ANOVA, ^∗∗∗^*p* < 0.001.

We confirm that *GPP2* is required for membrane and cell wall integrity as showed before (Jung et al., 2018). As expected, we observed that our mutants are more sensitive to Congo red and SDS (Figure 2A). In addition, we also found out that *gpp2*Δ strains are more sensitive to alkaline stress (Figure 2B). Growth of *gpp2* mutant was compared to wild type in the presence of hydrogen peroxide and only a slight difference was detected in YEPD. On SD, mutant and wild type were highly sensitive to even small concentrations of H_2_O_2_ (Supplementary Figure 2).

*Saccharomyces cerevisiae* Gpp2 is partially regulated by the Hog1 and is deeply involved in osmotic stress response (Norbeck et al., 1996; Pahlman et al., 2001). Therefore, we tested the hypothesis that *gpp2*Δ strains are hypersensitive to osmotic stress agents (NaCl and KCl) in rich YEPD and SD medium. As shown in Figure 2C, CNU125 and CNU126 have progressive growth impairment as the concentration of NaCl and KCl increases in YEPD and SD (0.75 and 1 M in YEPD and SD).

Since proline was able to rescue growth in SD as sole nitrogen source (Figure 1 and Table 1), we tested if it could also have any effect on growth rate in the presence of osmotic stress. As shown in Figure 2D, 20 mM proline as sole nitrogen source (but not 10 mM) was enough to relief the effects of 0.5 M of NaCl on growth in SD. The supplementation of glycerol was also sufficient to relief growth arrest caused by osmotic and cold shock (Figure 2E) in the *gpp2*Δ mutants. Since SD is a salt medium, it is possible that it represents an osmotic unbalanced condition for the *gpp2* mutant and that is the reason why 10 mM of proline was enough to overcome growth arrest in SD, but not in SD plus 0.5 M NaCl. An increase concentration of proline (20 mM) was necessary to overcome the osmotic stress in SD medium supplemented with 0.5 M of NaCl. Proline was also able to suppress some of the osmotic effects of NaCl in YEPD, but not comparable to SD, probably because amino acid transport is more efficient in SD than YEPD, due to relief of nitrogen catabolite repression on permeases (Fernandes et al., 2015).

These results reinforce the idea that this gene encodes the glycerol-3-phosphate phosphatase (*GPP2*), which is responsible for generating glycerol in *S. cerevisiae*, an important osmolyte that helps the cell to overcome osmotic and cold stress. In the absence of glycerol, *C. neoformans* can compensate osmotic unbalance with proline.

In addition to the osmotic and cold shock phenotypes associated to *gpp*2 mutant, we tested if the transcription of *GPP2* would be induced in the presence osmotic stress. Figure 2F shows that *GPP2* is induced 7.1 fold in YEPD + 1 M NaCl relative to YEPD in H99. This result and the phenotypic characterization strongly indicate that the glycerol phosphate phosphatase encoded by *GPP2* is involved in osmotic stress and probably is the homolog of *S. cerevisiae GPP2*.

### *GPP2* Is Important for Virulence in *C. neoformans*

**FIGURE 3:**
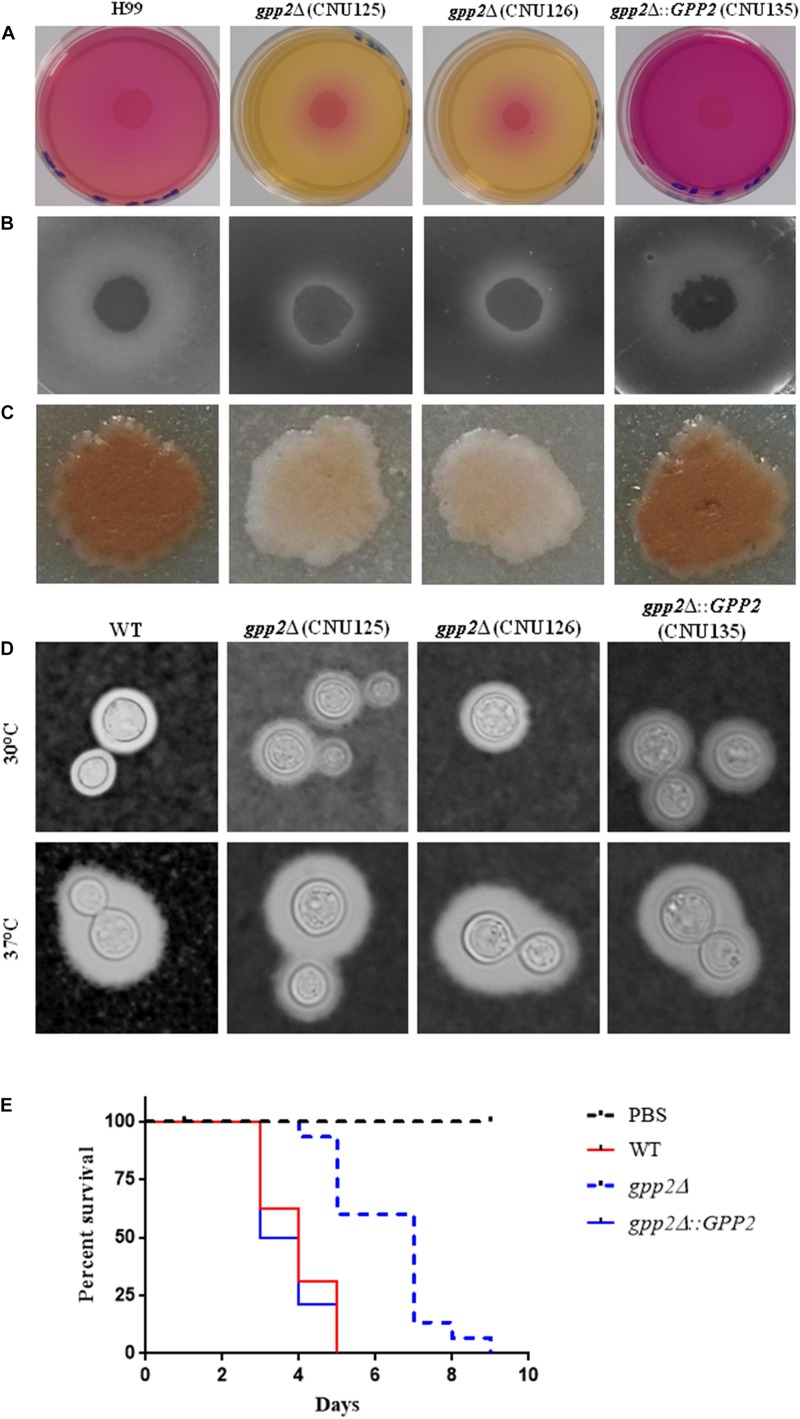
Virulence factor evaluation of the *gpp2* mutant compared to wild type and reconstituted strains. **(A)** urease, **(B)** phospholipase, **(C)** melanin, **(D)** capsule, and **(E)** invertebrate animal model in *G. mellonella*.

Regarding the virulence factors, the *gpp2*Δ strains were unable to produce urease (Figure 3A), phospholipase (Figure 3B) and melanin (Figure 3C) compared to wild type and the reconstituted strain. However, capsule production was not significantly different from wild type (Figure 3D). *gpp2*Δ strain (CNU125) was hypovirulent in *G. mellonella* animal model (Figure 3E) which is consistent with the data generated by Jung in mouse model of infection (Jung et al., 2018).

### Global Transcriptional Profile of the *gpp2*Δ Strain

**FIGURE 4:**
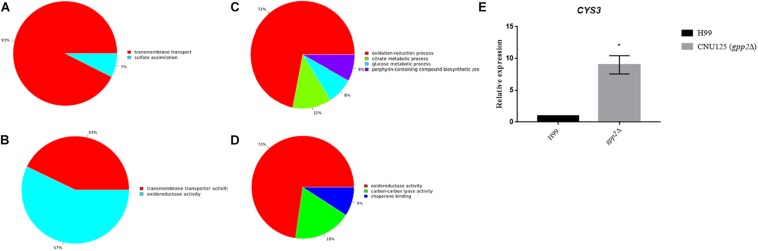
Graphs represent the DEGs found to be up **(A,B)** and down **(C,D)** regulated according to GO categories based on biological processes (**A** up regulated and **C** down regulated) and molecular function (**B** up regulated and **D** down regulated). The cutoff used was 1.0 Log2. **(E)** Graph shows *CYS3* gene quantification by Real time qPCR in gpp2Δ strain relative to wild type. RNA was extracted from cultures growing in YEPD for 2 h at 30°C.

In order to draw a broader picture on how *GPP2* affects cellular metabolism, amino acid transport/biosynthesis, stress and the sulfur amino acids biosynthesis, the global transcription profile of *gpp2*Δ strain was done by RNAseq and compared to wild type. As a validation control of the experiment we detected that CNAG_01744 (encoding *GPP2*) is not expressed in the mutant, as expected. A total of 205 DEGs (Differentially Expressed Genes) were up regulated and 129 were down regulated in the mutant relative to wild type (Supplementary Tables 2, 3) considering a 1.0 log2Fold change as cutoff. Among the DEGs listed, many encode hypothetical proteins (126 up regulated 66 down regulated). Transport and sulfate assimilation are the main biological processes and molecular functions pointed out by GO analysis that appeared as up regulated two fold or more (Table 2 and Figures 4A,B and Supplementary Table 2).

**TABLE 2:** List of the biological processes and molecular functions found to be differentially expressed (up and down regulated), according to Gene ontology (GO), in *gpp2*Δ mutant relative to wild type.

**Up regulated (log2(FC) > 1.0)**				

**GOID**	**GO term**	**Genes found**	**Size**	***q* value**
**Biological process**				
GO:0055085	Transmembrane transport	25	333	0.0
GO:0000103	Sulfate assimilation	6	15	0.002
**Molecular functions**				
GO:0022857	Transmembrane transporter activity	12	208	0.001
GO:0016491	Oxidoreductase activity	16	365	0.002

**Down regulated (log2(FC) < −1.0)**				

**GOID**	**GO term**	**Genes found**	**Size**	***q* value**

**Biological process**				
GO:0055114	Oxidation-reduction process	18	333	0.00
GO:0006101	Citrate metabolic process	3	16	0.002
GO:0006006	Glucose metabolic process	2	7	0.006
GO:0006778	Porphyrin-containing compound metabolic process	2	8	0.008
**Molecular functions**				
GO:0016491	Oxidoreductase activity	16	365	0.0
GO:0016830	Carbon-carbon lyase activity	4	4	0.0
GO:0051087	Chaperone binding	2	2	0.04

*The GO functions were enriched by the hypergeometric test with the cutoff of 0.01.*

Among the transporters, sulfite (CNAG_00529), pantothenate (CNAG_00540), myo-inositol transporters (CNAG_00864 and CNAG_00867), amino acids permeases (CNAG_00597, 00728 and 01118), ion (CNAG_00979), phospholipid (CNAG_01055), and sugar transporters (CNAG_01683, 03772, 04474, and 06963) are examples of the most induced genes found in this analysis. Also, a major facilitator transporter super family gene (MFS, CNAG_06610) was up regulated. If we consider less than two fold change (0.5 log2Fold change) the number of induced transporters rises to 70 DEGs up regulated on the same categories described above. CNAG_01683, which was found to be 1.16 fold induced (log2), encodes a protein that shares 49% of amino acid sequence similarity to *S. cerevisiae Stl1*, a glycerol/H^+^ symporter that is thought to play a major role on glycerol transport. Its transcription is induced in the presence of glycerol and osmotic shock and it is repressed on glucose (Ferreira et al., 2005). The up regulation of these transporter genes in the gpp2Δ strain relative to the wild type indicates that small molecule translocation, such as amino acids and inositol, may function as a compensatory mechanism to the lack of glycerol, an important osmolyte.

A total of six genes of sulfur amino acid biosynthetic pathway (CNAG_02202, 03898, 04215, 04798, 06448, 02270), plus a sulfite transporter (CNAG_00529) and the arylsulfatase (CNAG_01498) were also induced in the mutant. *MET17*, encoding the cysteine synthase (CNAG_05028), was induced, however, less than two log2Fold change (0.66 fold induction *p* = 2.20 × 10^e–08^). These results are in agreement with our published data, in which, the deletion of *GPP2* causes Cys3 protein to accumulate in higher levels compared to wild type, suggesting that Gpp2 is involved in the Cys3 degradation and consequently down regulation of the sulfur assimilation network (de Melo et al., 2019). In order to validate our transcriptome data regarding the up regulation of the sulfur uptake pathway in the *GPP2* mutant, we calculated the expression level of *CYS3* in CNU125 (gpp2Δ) by RT-qPCR. We found that Cys3 is up regulated nearly 8.9 fold in the mutant compared to wild type (Figure 4E). These results show that lack of *GPP2* not only modulate *CYS3* at the post translational levels, but also changes gene regulation on the sulfur assimilatory circuit.

Among the down regulated DEGs, most of them are related to oxidation and reduction reaction, tricarboxylic, glucose and antibiotic metabolism (Table 2 and Figures 4C,D and Supplementary Table 3). This result is consistent with the role of Gpp2 as an important phosphatase involved in glycerol biosynthesis, which is not only important for osmoregulation, but also it is a key player in glycolysis and tricarboxylic acid metabolism (Possik et al., 2017).

Genes related to oxidative stress response were differentially regulated in the *gpp2*Δ, among them, the most induced are: catalase 2 (CNAG_05256 3.0 log2 Fold change), mitochondrial cytochrome C peroxidase (CNAG_01138 2.12 log2 Fold change) and glutathione transferase (CNAG_03848, 1.16 log2 Fold change).

Interestingly, three taurine dioxygenases (TauD) were induced in the mutant (CNAG_01542 and CNAG_06249 and CNAG_06876 at 4.0, 0.91 and 0.72 log2Fold change, respectively). This result suggests that the deletion of *GPP2* not only derepresses sulfur uptake regulatory network through *CYS3* expression, but also may induce the mobilization of sulfite from sulfonates, such as taurine. In *S. cerevisiae*, *JLP1* is the only α-ketoglutarate dioxygenase known to release sulfite from isethionate and taurine (Hogan et al., 1999; Holt et al., 2017). Orthologs of *JLP1* are genome linked to a predicted sulfur/sulfonate transporter in filamentous *Eurotiomycete* and *Basidiomycota* (Kensche et al., 2008). In *S. cerevisiae* the sulfur/sulfonate transporter is encoded by Soa1 gene, however, this gene pairing arrangement was lost in Ascomycetes (Holt et al., 2017). We analyzed if this genomic organization would be present in *C. neoformans* as well. Indeed, on chromosome 11 the arylsulfatase (CNAG_01498), sulfonate/sulfur transporter (CNAG_01499) and two taurine dioxygenases (CNAG_01500 and CNAG_01542) are clustered. Except for CNAG_01500, all other genes in this cluster are up regulated in the *gpp2*Δ strain.

It is important to mention that CNAG_01501, which is located between the two taurine dioxygenases genes, encodes a gamma-glutamyl cycle transferase (ChaC) involved in the biosynthesis of 5-oxoproline, an intermediate in the glutathione cycle (Kumar et al., 2012). This gene is not differentially regulated in *gpp2*Δ, but taken together these results suggests a connection between the osmotic stress, sulfur uptake and the glutathione cycle.

### Amino Acid Permeases Regulation in *gpp2*Δ Strain

**FIGURE 5:**
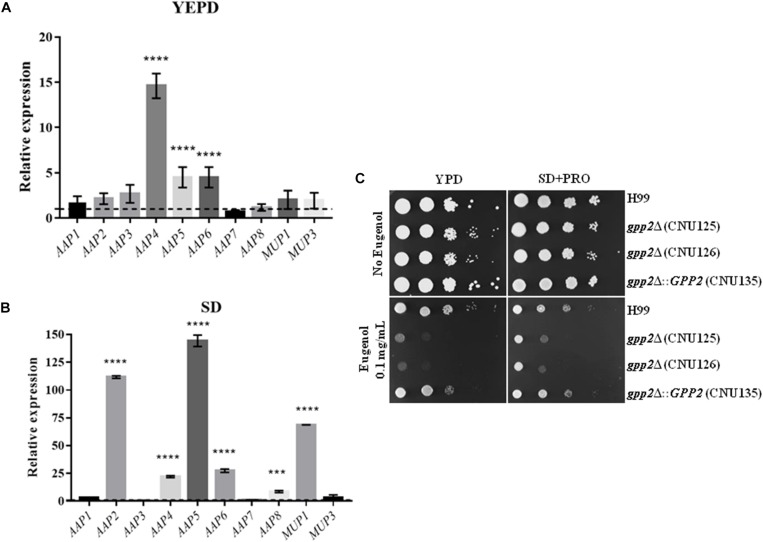
Expression of permease genes in the *gpp2*Δ mutant relative to wild type in **(A)** YEPD and **(B)** SD during nitrogen starvation by qPCR. Dotted line represents the expression level of the reference strain (wild type). Statistical significant differences were tested by Two-way ANOVA, ^∗∗∗∗^*p* < 0.0001 and ^∗∗∗^*p* < 0.001. **(C)** Growth of wild type, reconstituted (CNU135) and mutant strains (CNU125 and CNU 126) in YEPD without and with 0.1 g/L of eugenol (left panel) and the same strains in SD plus proline without and with 0.1 g/L eugenol (right panel).

These results prompted us to validate the expression profile of the amino acid permeases in the *gpp2* mutant. qPCR was applied to quantify the expression of the *AAP* genes, *MUP1*, and *MUP3* in YEPD and during nitrogen starvation. The graph in Figure 5A shows that permease genes *AAP4*, *AAP5*, and *AAP6* are significantly induced. During nitrogen starvation the expression level, in general, was higher compared to YEPD; notably permeases *AAP2, AAP4, AAP5, AAP6, AAP8*, and *MUP1* had the highest induction (Figures 5A,B). The higher expression in SD compared to YEPD is due to the removal of nitrogen catabolite repression, which is known to induce permease gene expression (Fernandes et al., 2015; Martho et al., 2016; Calvete et al., 2019).

Earlier, our group and other identified the amino acid permeases as the target of Eugenol, a natural product extracted from cloves that has antimicrobial activity (Darvishi et al., 2013; Martho et al., 2016). The deletion of permeases *AAP4* and *AAP5* renders *C. neoformans* resistant to eugenol, therefore, we reason that the up regulation of permease genes in *gpp2*Δ strain would confer sensitivity to eugenol, since its targets are over represented. As shown in Figure 5C, the gpp2Δ mutants are more sensitive to eugenol than wild type and reconstituted strains, as we predicted. The addition of proline to the medium was not able to compete with eugenol and promote a better growth.

**FIGURE 6:**
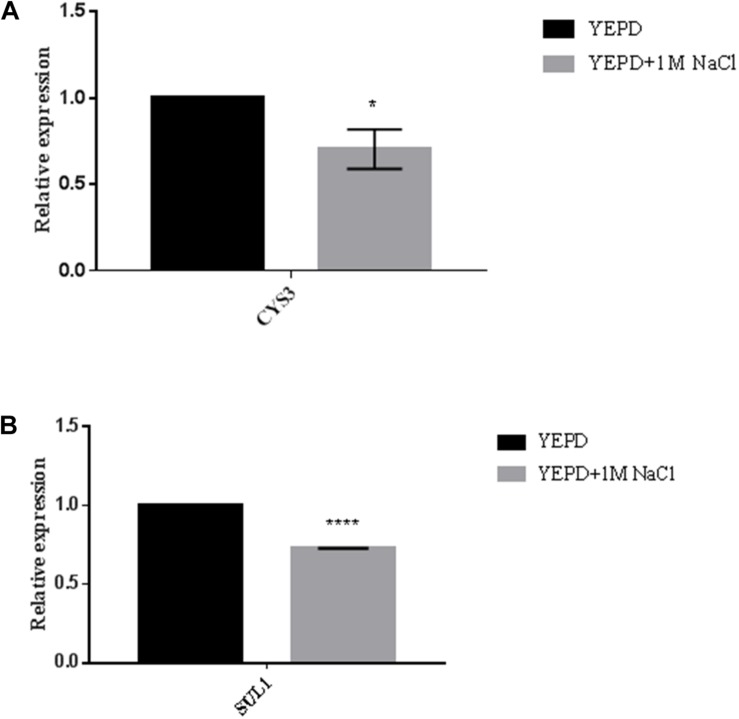
Expression pattern of *CYS3* **(A)** and *SUL1* **(B)** in wild type strain under osmotic stress by qPCR. YEPD was supplemented with 1 M of NaCl. Statistical significant differences were tested by Two-way ANOVA, ^∗∗∗∗^*p* < 0.0001 and ^∗^*p* < 0.05.

At this point our data suggest that *GPP2* is essential for glycerol generation, which seems to be the main osmolyte in *C. neoformans*. Without glycerol, cells have to uptake amino acids to achieve osmotic balance. The RNAseq data also suggest that lack of glycerol biosynthesis, and consequently the inability to cope even with small amounts of osmotic stress (SD medium), leads to activation of sulfur amino acid biosynthesis and oxidative stress response. These two metabolic pathways are deeply connected, since cysteine feeds the glutathione cycle, which is required to counter act oxidative stress. Since glycerol is the main osmolyte, sulfur amino acid biosynthesis and glutathione cycle would be required only in the absence of glycerol biosynthesis such as in the *gpp2*Δ strain. It is possible that sulfur amino acid and glutathione biosynthesis are not induced in the presence of osmotic stress, as long as glycerol is available in the cell. In order to test this hypothesis the expression levels of *CYS3* and *SUL1* were calculated in the presence of 1 M of NaCl in wild type. Interestingly, the expression levels of *CYS3* and *SUL1* were slightly down regulated (30% each) in the presence of osmotic stress as shown in Figures 6A,B, respectively, suggesting that, in fact, sulfur uptake network may be required only if glycerol is not available to counteract osmotic stress. However, further investigation will be necessary develop and confirm this hypothesis.

## Discussion

The ability to adapt to sudden changes is a challenge encountered by many living organisms, specially microbes and plants. Since fungal pathogens occupy many different niches, they are vulnerable to nutritional, oxidative, cell wall and various types of stresses (Kronstad et al., 2012). Several of the genetic circuits which are triggered by these signals cross talk to each other eliciting responses that regulate many different genes producing a physiological conditions that allows survival even in most adverse conditions (Lin and Heitman, 2006; Brown et al., 2007; Lin, 2009; Li and Mody, 2010).

In this paper, we have explored several phenotypes of *C. neoformans* survival and virulence associated to the deletion of the glycerol-3-phospate phosphatase, which is responsible to carry the last step in the biosynthesis of glycerol, a major cellular osmolyte that helps the cells to counter act the effects of osmotic and cold shock (Norbeck et al., 1996). Also, we identified a link between *GPP2* and the transcription of the sulfur amino acid biosynthetic pathway.

*C. neoformans* encodes 4 proteins with features of the Halo Acid Dehalogenases (HAD). In our previous work we identified one of them (CNAG_01744) and named it as *GPP2* (de Melo et al., 2019). Among other reasons, this gene was designated so, due to its role on osmotic stress, which resembles phenotypes related to *S. cerevisiae GPP2*. Jung et al., also studied *GPP2*, but named it as *HAD1* and described it as a genetic element involved in cell wall integrity (Jung et al., 2018); however, in *S. cerevisiae HAD1* gene encodes an enzyme enrolled in the biosynthesis of nicotinic acid. Therefore, we maintained the name of the gene as *GPP2* to avoid confusion.

Besides being transcriptionally induced by 1 M of NaCl, *GPP2* is essential to counteract osmotic stress, membrane, cell wall and alkaline stresses. In our work we identified *GPP2* as primordial to tolerate cold shock (Figures 1, 2). In *S. cerevisiae* it is well know that the Hog pathway is responsible for coordinating cold shock response (Hayashi and Maeda, 2006; Panadero et al., 2006). Ko and collaborators identified *GPP2* as a target of Hog1 pathway (Ko et al., 2009) in *C. neoformans*. In this manuscript, we report for the first time a mutant that is sensitive to cold and osmotic stresses.

Another interesting feature of the *gpp2*Δ mutant is the low growth rate in SD in the presence of the preferred nitrogen source (ammonium sulfate), suggesting a nutritional defect. Curiously, growth arrest could be reverted by several single amino acids, as sole nitrogen source (Figure 2 and Table 1). One explanation for the observation is that certain amino acids improve growth rates in the *gpp2*Δ strains because they serve as osmolytes that compensate the lack of glycerol. As shown in this paper, proline was able to revert osmotic shock caused by NaCl confirming that this amino acids act as compatible solute in *C. neoformans gpp2*Δ mutant. In bacteria, archaea, and eukarya, proline and other amino acids function as compatible solute, protecting the cells against the osmotic stress (Kempf and Bremer, 1998).

The global transcriptional profile showed that transmembrane transport of several substrates into the cell is up regulated in the *gpp2*Δ. Among these transporters, amino acid permeases are highly induced (Figures 4, 5), suggesting that indeed amino acids may, at least partially, contribute to counteract osmotic stress. This result is in agreement with a previous transcriptome analysis reported in the literature in which amino acid permeases are induced in a Hog1 mutant (Ko et al., 2009). Also, the positive role of amino acids on osmotic stress is well known in many microorganisms and plants (Peddie et al., 1994; Zaprasis et al., 2015; Zou et al., 2016) The fact that amino acids rescued growth and permeases are induced in the *gpp2* mutant argues that amino acids are important compatible solutes that combat osmotic stress in *C. neoformans*.

**FIGURE 7:**
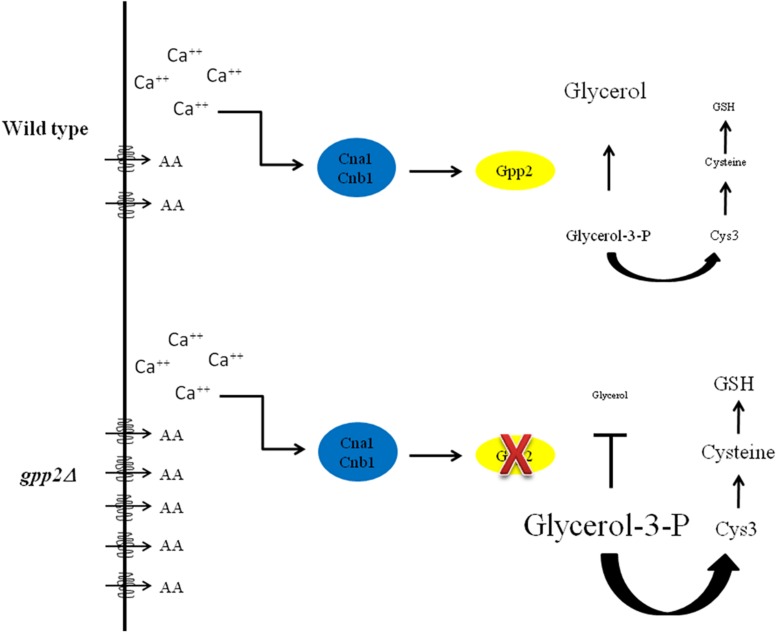
Model representing the main findings in this work. AA = amino acids; transmembrane drawing = amino acid permeases; Cna1 and Cnb1 = calcineurin catalytic and regulatory subunits respectively; GSH = glutathione.

These results suggest that there is a link between osmotic stress, sulfur amino acid biosynthesis and amino acid uptake, which is depicted as a model in Figure 7. Since some amino acids can be considered osmolytes, it makes sense that an interruption on the biosynthesis of an important osmoregulator (glycerol) may activate the biosynthesis and uptake of alternative molecules that could serve as osmolytes, as an attempt to provide osmobalance to the cell. One possibility is that, accumulation of the Gpp2 substrate (glycerol-3-phosphate) signals the biosynthesis or uptake of other substances that promote osmotic balance, such as amino acids, which are capable of bypassing the growth arrest caused by osmotic shock in the *gpp2* mutant (Figure 7).

The transcriptome analysis showed that oxidative stress response may be active in the *gpp2*Δ. In support of this finding, Ko and collaborators (Ko et al., 2009) detected an increase in the expression of genes related to this type of stress in *C. neoformans hog1*Δ. Also, in *S. cerevisiae*, *GPP1*, and *GPP2* are both involved in oxidative stress response, in addition to osmotic stress (Pahlman et al., 2001). Osmotic, nutritional and oxidative conditions are diverse signals which trigger specific stress response pathways; however, one signal may elicit more than one stress response pathway (Taymaz-Nikerel et al., 2016). Often, osmotic stress signals lead to osmotic and oxidative stress responses, exactly as we see in this case; one interpretation is that osmotic stress also generates oxidative stress, which must be mitigated for survival (Figure 7).

Our previous data and the present one showed that *GPP2* is important for Cys3 protein modulation (de Melo et al., 2019) and allowed us to assemble a model to explain the facts observed here (Figure 7). In this paper, we provided further evidence that *gpp2* deletion caused an up regulation of several genes in the sulfur uptake pathway and sulfur amino acid biosynthesis. Ko and collaborators also found two genes up regulated in this pathway in a *hog1* deletion mutant (Ko et al., 2009). It is possible that up regulation of the sulfur uptake pathway leads to cysteine increase which would reflect in a higher ability to counter act oxidative stress through glutathione synthesis (Figure 7). The fact that taurine dioxygenases are up regulated indicate that sulfite is been generated from sulfonate and could increase the cysteine pools and consequently glutathione. In plants, the generation of intermediate metabolites in the sulfur amino acid biosynthetic routes, especially hydrogen sulfite, alleviates osmotic and oxidative stress by accumulation of osmolytes and synthesis of antioxidant enzymes (Anjum et al., 2017; Khan et al., 2017; Zhou et al., 2018). In fact, the sulfur metabolism has been considered as a defense system against biotic and abiotic stresses in plants (Rausch and Wachter, 2005).

In this paper, we revealed that *GPP2* is (i) involved in osmotic stress response, (ii) is required for cold shock and transcriptionally induced by salt; (iii) it affects the expression of amino acid permeases and oxidative stress genes, and (v) it also influences the sulfur amino acid biosynthetic pathway. Cell wall integrity and ability to cope with alkaline condition are also affected by *GPP2* function. Expression of virulence factors was highly reduced by *GPP2* deletion, which in conjunction with the inability to cope with stresses, led to hypovirulence in *G. mellonella* animal model. The connections found in this paper between *GPP2*, the various types of stress and its link with the sulfur metabolism, which is a metabolic process that has been well proved to be linked to virulence, open a window of opportunities for therapeutical possibilities in pathogenic fungi.

## Data Availability Statement

The raw data supporting the conclusions of this manuscript will be made available by the authors, without undue reservation, to any qualified researcher.

## Author Contributions

KM performed the data collection and analysis. AV and OB performed the bioinformatics analysis (RNAseq). MV provided the financial support and wrote the manuscript. RP contributed with financial support, study design, data analysis, and manuscript writing and editing.

## Conflict of Interest

The authors declare that the research was conducted in the absence of any commercial or financial relationships that could be construed as a potential conflict of interest.

## Footnotes

1 https://jgi.doe.gov/data-and-tools/bbtools/bb-tools-user-guide/bbduk-guide

2 https://www.bioinformatics.babraham.ac.uk/projects/fastqc

3 https://fungidb.org

## References

[B1] Albertyn J. Hohmann S. Thevelein J. M. Prior B. A. (1994). GPD1, which encodes glycerol-3-phosphate dehydrogenase, is essential for growth under osmotic stress in *Saccharomyces cerevisiae*, and its expression is regulated by the high-osmolarity glycerol response pathway. *Mol. Cell Biol.* 14 4135–4144. 10.1128/mcb.14.6.4135 8196651 PMC358779

[B2] Anjum S. A. Ashraf U. Tanveer M. Khan I. Hussain S. Shahzad B. (2017). Drought induced changes in growth, osmolyte accumulation and antioxidant metabolism of three maize hybrids. *Front. Plant Sci.* 8:69. 10.3389/fpls.2017.00069 28220130 PMC5292435

[B3] Ballou E. R. Johnston S. A. (2017). The cause and effect of cryptococcus interactions with the host. *Curr. Opin. Microbiol.* 40 88–94. 10.1016/j.mib.2017.10.012 29154043

[B4] Brown S. M. Campbell L. T. Lodge J. K. (2007). *Cryptococcus neoformans*, a fungus under stress. *Curr. Opin. Microbiol.* 10 320–325. 10.1016/j.mib.2007.05.014 17707685 PMC2570326

[B5] Calvete C. L. Martho K. F. Felizardo G. Paes A. Nunes J. M. Ferreira C. O. (2019). Amino acid permeases in *Cryptococcus neoformans* are required for high temperature growth and virulence; and are regulated by Ras signaling. *PLoS One* 14:e0211393. 10.1371/journal.pone.0211393 30682168 PMC6347259

[B6] Christensen W. B. (1946). Urea decomposition as a means of differentiating proteus and paracolon cultures from each other and from *Salmonella* and *Shigella* Types. *J. Bacteriol.* 52 461–466. 16561200 10.1128/jb.52.4.461-466.1946 PMC518212

[B7] Cunningham K. W. Fink G. R. (1994). Calcineurin-dependent growth control in Saccharomyces cerevisiae mutants lacking PMC1, a homolog of plasma membrane Ca2+ ATPases. *J. Cell Biol.* 124 351–363. 10.1083/jcb.124.3.351 7507493 PMC2119937

[B8] Darvishi E. Omidi M. Bushehri A. A. Golshani A. Smith M. L. (2013). The antifungal eugenol perturbs dual aromatic and branched-chain amino acid permeases in the cytoplasmic membrane of yeast. *PLoS One* 8:e76028. 10.1371/journal.pone.0076028 24204588 PMC3799837

[B9] de Melo A. T. Martho K. F. Roberto T. N. Nishiduka E. S. Machado J. Brustolini O. J. B. (2019). The regulation of the sulfur amino acid biosynthetic pathway in *Cryptococcus neoformans*: the relationship of Cys3, calcineurin, and Gpp2 phosphatases. *Sci. Rep.* 9:11923. 10.1038/s41598-019-48433-5 31417135 PMC6695392

[B10] Dobin A. Davis C. A. Schlesinger F. Drenkow J. Zaleski C. Jha S. (2013). STAR: ultrafast universal RNA-seq aligner. *Bioinformatics* 29 15–21. 10.1093/bioinformatics/bts635 23104886 PMC3530905

[B11] Esher S. K. Zaragoza O. Alspaugh J. A. (2018). Cryptococcal pathogenic mechanisms: a dangerous trip from the environment to the brain. *Mem. Inst. Oswaldo Cruz* 113:e180057. 10.1590/0074-02760180057 29668825 PMC5909089

[B12] Falcon S. Gentleman R. (2007). Using GOstats to test gene lists for GO term association. *Bioinformatics* 23 257–258. 10.1093/bioinformatics/btl567 17098774

[B13] Fan W. Idnurm A. Breger J. Mylonakis E. Heitman J. (2007). Eca1, a sarcoplasmic/endoplasmic reticulum Ca2+-ATPase, is involved in stress tolerance and virulence in *Cryptococcus neoformans*. *Infect. Immun.* 75 3394–3405. 10.1128/iai.01977-06 17502401 PMC1932933

[B14] Fernandes J. D. Martho K. Tofik V. Vallim M. A. Pascon R. C. (2015). The role of amino acid permeases and tryptophan biosynthesis in *Cryptococcus neoformans* Survival. *PLoS One* 10:e0132369. 10.1371/journal.pone.0132369 26162077 PMC4498599

[B15] Ferreira C. van F. Voorst, Martins A. Neves L. Oliveira R. (2005). A member of the sugar transporter family, Stl1p is the glycerol/H+ symporter in *Saccharomyces cerevisiae*. *Mol. Biol. Cell* 16 2068–2076. 10.1091/mbc.e04-10-0884 15703210 PMC1073684

[B16] Garcia-Vidal C. Viasus D. Carratala J. (2013). Pathogenesis of invasive fungal infections. *Curr. Opin. Infect. Dis.* 26 270–276. 10.1097/QCO.0b013e32835fb920 23449139

[B17] Hayashi M. Maeda T. (2006). Activation of the HOG pathway upon cold stress in *Saccharomyces cerevisiae*. *J. Biochem.* 139 797–803. 10.1093/jb/mvj089 16672281

[B18] Hogan D. A. Auchtung T. A. Hausinger R. P. (1999). Cloning and characterization of a sulfonate/alpha-ketoglutarate dioxygenase from *Saccharomyces cerevisiae*. *J. Bacteriol.* 181 5876–5879. 10482536 10.1128/jb.181.18.5876-5879.1999 PMC94115

[B19] Holt S. Kankipati H. De Graeve S. Van Zeebroeck G. Foulquie-Moreno M. R. Lindgreen S. (2017). Major sulfonate transporter Soa1 in *Saccharomyces cerevisiae* and considerable substrate diversity in its fungal family. *Nat. Commun.* 8:14247. 10.1038/ncomms14247 28165463 PMC5303821

[B20] Idnurm A. Reedy J. L. Nussbaum J. C. Heitman J. (2004). Cryptococcus neoformans virulence gene discovery through insertional mutagenesis. *Eukaryot Cell* 3 420–429. 10.1128/ec.3.2.420-429.2004 15075272 PMC387659

[B21] Jung W. H. Son Y. E. Oh S. H. Fu C. Kim H. S. Kwak J. H. (2018). Had1 is required for cell wall integrity and fungal virulence in *Cryptococcus neoformans*. *G3* 8 643–652. 10.1534/g3.117.300444 29233914 PMC5919746

[B22] Kempf B. Bremer E. (1998). Uptake and synthesis of compatible solutes as microbial stress responses to high-osmolality environments. *Arch. Microbiol.* 170 319–330. 9818351 10.1007/s002030050649

[B23] Kensche P. R. Oti M. Dutilh B. E. Huynen M. A. (2008). Conservation of divergent transcription in fungi. *Trends Genet.* 24 207–211. 10.1016/j.tig.2008.02.003 18375009

[B24] Khan M. N. Mobin M. Abbas Z. K. Siddiqui M. H. (2017). Nitric oxide-induced synthesis of hydrogen sulfide alleviates osmotic stress in wheat seedlings through sustaining antioxidant enzymes, osmolyte accumulation and cysteine homeostasis. *Nitric Oxide* 68 91–102. 10.1016/j.niox.2017.01.001 28062279

[B25] Ko Y. J. Yu Y. M. Kim G. B. Lee G. W. Maeng P. J. Kim S. (2009). Remodeling of global transcription patterns of *Cryptococcus neoformans* genes mediated by the stress-activated HOG signaling pathways. *Eukaryot Cell* 8 1197–1217. 10.1128/EC.00120-09 19542307 PMC2725552

[B26] Kraus P. R. Heitman J. (2003). Coping with stress: calmodulin and calcineurin in model and pathogenic fungi. *Biochem. Biophys. Res. Commun.* 311 1151–1157. 10.1016/s0006-291x(03)01528-6 14623301

[B27] Kronstad J. Saikia S. Nielson E. D. Kretschmer M. Jung W. Hu G. (2012). Adaptation of *Cryptococcus neoformans* to mammalian hosts: integrated regulation of metabolism and virulence. *Eukaryot Cell* 11 109–118. 10.1128/EC.05273-11 22140231 PMC3272904

[B28] Kumar A. Tikoo S. Maity S. Sengupta S. Sengupta S. Kaur A. (2012). Mammalian proapoptotic factor ChaC1 and its homologues function as gamma-glutamyl cyclotransferases acting specifically on glutathione. *EMBO Rep.* 13 1095–1101. 10.1038/embor.2012.156 23070364 PMC3512401

[B29] Larsson K. Ansell R. Eriksson P. Adler L. (1993). A gene encoding sn-glycerol 3-phosphate dehydrogenase (n.d.) complements an osmosensitive mutant of *Saccharomyces cerevisiae*. *Mol. Microbiol.* 10 1101–1111. 10.1111/j.1365-2958.1993.tb00980.x 7934860

[B30] Li S. S. Mody C. H. (2010). Cryptococcus. *Proc. Am. Thorac. Soc.* 7 186–196. 10.1513/pats.200907-063AL 20463247

[B31] Liao Y. Smyth G. K. Shi W. (2019). The R package rsubread is easier, faster, cheaper and better for alignment and quantification of RNA sequencing reads. *Nucleic Acids Res.* 47:e47. 10.1093/nar/gkz114 30783653 PMC6486549

[B32] Limper A. H. Adenis A. Le T. Harrison T. S. (2017). Fungal infections in HIV/AIDS. *Lancet Infect. Dis.* 17 e334–e343. 10.1016/S1473-3099(17)30303-1 28774701

[B33] Lin X. (2009). *Cryptococcus neoformans*: morphogenesis, infection, and evolution. *Infect. Genet. Evol.* 9 401–416. 10.1016/j.meegid.2009.01.013 19460306

[B34] Lin X. Heitman J. (2006). The biology of the *Cryptococcus neoformans* species complex. *Annu. Rev. Microbiol.* 60 69–105. 16704346 10.1146/annurev.micro.60.080805.142102

[B35] Livak K. J. Schmittgen T. D. (2001). Analysis of relative gene expression data using real-time quantitative PCR and the 2(-Delta Delta C(T)) Method. *Methods* 25 402–408. 10.1006/meth.2001.1262 11846609

[B36] Love M. I. Huber W. Anders S. (2014). Moderated estimation of fold change and dispersion for RNA-seq data with DESeq2. *Genome. Biol.* 15:550. 25516281 10.1186/s13059-014-0550-8 PMC4302049

[B37] Luo W. Brouwer C. (2013). Pathview: an R/bioconductor package for pathway-based data integration and visualization. *Bioinformatics* 29 1830–1831. 10.1093/bioinformatics/btt285 23740750 PMC3702256

[B38] Martho K. F. de Melo A. T. Takahashi J. P. Guerra J. M. Santos D. C. Purisco S. U. (2016). Amino acid permeases and virulence in *Cryptococcus neoformans*. *PLoS One* 11:e0163919. 10.1371/journal.pone.0163919 27695080 PMC5047642

[B39] Mylonakis E. Moreno R. El Khoury J. B. Idnurm A. Heitman J. Calderwood S. B. (2005). Galleria mellonella as a model system to study *Cryptococcus neoformans* pathogenesis. *Infect. Immun.* 73 3842–3850. 10.1128/iai.73.7.3842-3850.2005 15972469 PMC1168598

[B40] Norbeck J. Pahlman A. K. Akhtar N. Blomberg A. Adler L. (1996). Purification and characterization of two isoenzymes of DL-glycerol-3-phosphatase from *Saccharomyces cerevisiae*. identification of the corresponding GPP1 and GPP2 genes and evidence for osmotic regulation of Gpp2p expression by the osmosensing mitogen-activated protein kinase signal transduction pathway. *J. Biol. Chem.* 271 13875–13881. 10.1074/jbc.271.23.13875 8662716

[B41] Pahlman A. K. Granath K. Ansell R. Hohmann S. Adler L. (2001). The yeast glycerol 3-phosphatases Gpp1p and Gpp2p are required for glycerol biosynthesis and differentially involved in the cellular responses to osmotic, anaerobic, and oxidative stress. *J. Biol. Chem.* 276 3555–3563. 10.1074/jbc.m007164200 11058591

[B42] Paliwal D. K. Randhawa H. S. (1978). A rapid pigmentation test for identification of *Cryptococcus neoformans*. *Antonie Van Leeuwenhoek* 44 243–246. 10.1007/bf00643226 109041

[B43] Panadero J. Pallotti C. Rodriguez-Vargas S. Randez-Gil F. Prieto J. A. (2006). A downshift in temperature activates the high osmolarity glycerol (HOG) pathway, which determines freeze tolerance in *Saccharomyces cerevisiae*. *J. Biol. Chem.* 281 4638–4645. 10.1074/jbc.m512736200 16371351

[B44] Park H. S. Chow E. W. Fu C. Soderblom E. J. Moseley M. A. Heitman J. (2016). Calcineurin targets involved in stress survival and fungal virulence. *PLoS Pathog.* 12:e1005873. 10.1371/journal.ppat.1005873 27611567 PMC5017699

[B45] Pascon R. C. Ganous T. M. Kingsbury J. M. Cox G. M. McCusker J. H. (2004). *Cryptococcus neoformans* methionine synthase: expression analysis and requirement for virulence. *Microbiology* 150 3013–3023. 10.1099/mic.0.27235-0 15347759

[B46] Peddie B. A. Lever M. Hayman C. M. Randall K. Chambers S. T. (1994). Relationship between osmoprotection and the structure and intracellular accumulation of betaines by *Escherichia coli*. *FEMS Microbiol. Lett.* 120 125–131. 10.1016/0378-1097(94)00188-x 8056284

[B47] Possik E. Madiraju S. R. M. Prentki M. (2017). Glycerol-3-phosphate phosphatase/PGP: role in intermediary metabolism and target for cardiometabolic diseases. *Biochimie* 143 18–28. 10.1016/j.biochi.2017.08.001 28826615

[B48] Powderly W. G. (1993). Cryptococcal meningitis and AIDS. *Clin. Infect. Dis.* 17 837–842. 8286622 10.1093/clinids/17.5.837

[B49] Price M. F. Wilkinson I. D. Gentry L. O. (1982). Plate method for detection of phospholipase activity in Candida albicans. *Sabouraudia* 20 7–14. 10.1080/00362178285380031 7038928

[B50] Rausch T. Wachter A. (2005). Sulfur metabolism: a versatile platform for launching defence operations. *Trends Plant. Sci.* 10 503–509. 10.1016/j.tplants.2005.08.006 16143557

[B51] Rutherford J. C. Bahn Y. S. van den Berg B. Heitman J. Xue C. (2019). Nutrient and stress sensing in pathogenic yeasts. *Front. Microbiol.* 10:442. 10.3389/fmicb.2019.00442 30930866 PMC6423903

[B52] Saxena A. Sitaraman R. (2016). Osmoregulation in *Saccharomyces cerevisiae* via mechanisms other than the high-osmolarity glycerol pathway. *Microbiology* 162 1511–1526. 10.1099/mic.0.000360 27557593

[B53] Szklarczyk D. Morris J. H. Cook H. Kuhn M. Wyder S. Simonovic M. (2017). The STRING database in 2017: quality-controlled protein-protein association networks, made broadly accessible. *Nucleic Acids Res.* 45 D362–D368. 10.1093/nar/gkw937 27924014 PMC5210637

[B54] Taymaz-Nikerel H. Cankorur-Cetinkaya A. Kirdar B. (2016). Genome-wide transcriptional response of saccharomyces cerevisiae to stress-induced perturbations. *Front. Bioeng. Biotechnol.* 4:17. 10.3389/fbioe.2016.00017 26925399 PMC4757645

[B55] Toffaletti D. L. Rude T. H. Johnston S. A. Durack D. T. Perfect J. R. (1993). Gene transfer in *Cryptococcus neoformans* by use of biolistic delivery of DNA. *J. Bacteriol.* 175 1405–1411. 10.1128/jb.175.5.1405-1411.1993 8444802 PMC193227

[B56] Yang Z. Pascon R. C. Alspaugh A. Cox G. M. McCusker J. H. (2002). Molecular and genetic analysis of the *Cryptococcus neoformans* MET3 gene and a met3 mutant. *Microbiology* 148 2617–2625. 10.1099/00221287-148-8-2617 12177356

[B57] Zaprasis A. Bleisteiner M. Kerres A. Hoffmann T. Bremer E. (2015). Uptake of amino acids and their metabolic conversion into the compatible solute proline confers osmoprotection to *Bacillus subtilis*. *Appl. Environ. Microbiol.* 81 250–259. 10.1128/AEM.02797-14 25344233 PMC4272716

[B58] Zaragoza O. Casadevall A. (2004). Experimental modulation of capsule size in *Cryptococcus neoformans*. *Biol. Proc. Online* 6 10–15. 10.1251/bpo68 15103395 PMC389900

[B59] Zhou Z. H. Wang Y. Ye X. Y. Li Z. G. (2018). Signaling molecule hydrogen sulfide improves seed germination and seedling growth of maize (Zea mays L.) under high temperature by inducing antioxidant system and osmolyte biosynthesis. *Front. Plant Sci.* 9:1288. 10.3389/fpls.2018.01288 30233625 PMC6131983

[B60] Zhu A. Ibrahim J. G. Love M. I. (2018). Heavy-tailed prior distributions for sequence count data: removing the noise and preserving large differences. *Bioinformatics* 35 2084–2092. 10.1093/bioinformatics/bty895 30395178 PMC6581436

[B61] Zou H. Chen N. Shi M. Xian M. Song Y. Liu J. (2016). The metabolism and biotechnological application of betaine in microorganism. *Appl. Microbiol. Biotechnol.* 100 3865–3876. 10.1007/s00253-016-7462-3 27005411

