# Supplementary material for: The Glycerol Phosphatase Gpp2: A Link to Osmotic Stress, Sulfur Assimilation and Virulence in Cryptococcus neoformans
Source: Front Microbiol. 2019 Nov 26;10:2728. doi: 10.3389/fmicb.2019.02728 (PMC6901960; doi:10.3389/fmicb.2019.02728)
Supplement: Supplementary file 1 [file Table_1.DOCX]

Supplementary Table 1: Primers list

| **Primer Code** | **Sequence** | **F or R** | **Use** |
| --- | --- | --- | --- |
| PRCP185 | GCCTTATGGTATACTCTATGATG | F | qPCR AAP1 |
| PRCP186 | CCGTATGCCCGAGCACCGAGG | R | qPCR AAP1 |
| PRCP187 | CATGGTATACGCGATGATGG | F | qPCR AAP2 |
| PRCP188 | TCTGGCTCCCCAGAAGTTAATG | R | qPCR AAP2 |
| PRCP189 | TCTAACCATTCTTGGTATCG | F | qPCR AAP3 |
| PRCP190 | ATGTACCACCGAGATAAAAG | R | qPCR AAP3 |
| PRCP191 | CGAGGCAAAGAACCCACG | F | qPCR AAP4 |
| PRCP192 | AATCAAGATGCAAGCGTTTATG | R | qPCR AAP4 |
| PRCP193 | ACTTACTTGGACCTCTATCCTC | F | qPCR AAP5 |
| PRCP194 | TTTTCGGATCAGCTTGAAACC | R | qPCR AAP5 |
| PRCP195 | CCTTGAAAGACCGTTTCGGC | F | qPCR AAP6 |
| PRCP196 | TGTCACAAGTGTTGGGTCATTG | R | qPCR AAP6 |
| PRCP197 | TTACATCATTTCTGCTGTGTTC | F | qPCR AAP7 |
| PRCP198 | CATGTATGTGAAAGCGATGG | R | qPCR AAP7 |
| PRCP199 | TCTCTTTCTAGGGATTCTTATC | F | qPCR AAP8 |
| PRCP200 | CTCCGCCATATGGGCAGAAGC | R | qPCR AAP8 |
| PRCP246 | GGGCCATTCGTCTACCACTG | F | qPCR CYS3 |
| PRCP247 | TTCGTCGCTTGTCCTCCTCG | R | qPCR CYS3 |
| PRCP284 | GTTCAACTTCATTGTTGCCC | F | qPCR MUP1 |
| PRCP285 | AGGAACACGATCCCTAGTC | R | qPCR MUP1 |
| PRCP286 | TATGTGGCAGGCGAAATGC | F | qPCR MUP3 |
| PRCP287 | CCTTGCAGTTGTGTACAATC | R | qPCR MUP3 |
| PRCP400 | CCGCAAATACCAAACATCATCc | F | Wild type GPP2 |
| PRCP405 | GCCCACGTATTGTACTTCGTTC | R | Wild type GPP2 |
| PRCP466 | CATTCTCAGATCTGCTCACG | F | qPCR GPP2 |
| PRCP467 | GAAATATGTTGAGCTAGTGC | R | qPCR GPP2 |
