# Supplementary material for: The Glycerol Phosphatase Gpp2: A Link to Osmotic Stress, Sulfur Assimilation and Virulence in Cryptococcus neoformans
Source: Front Microbiol. 2019 Nov 26;10:2728. doi: 10.3389/fmicb.2019.02728 (PMC6901960; doi:10.3389/fmicb.2019.02728)
Supplement: Supplementary file 2 [file Presentation_1.PPTX]

## Slide 1
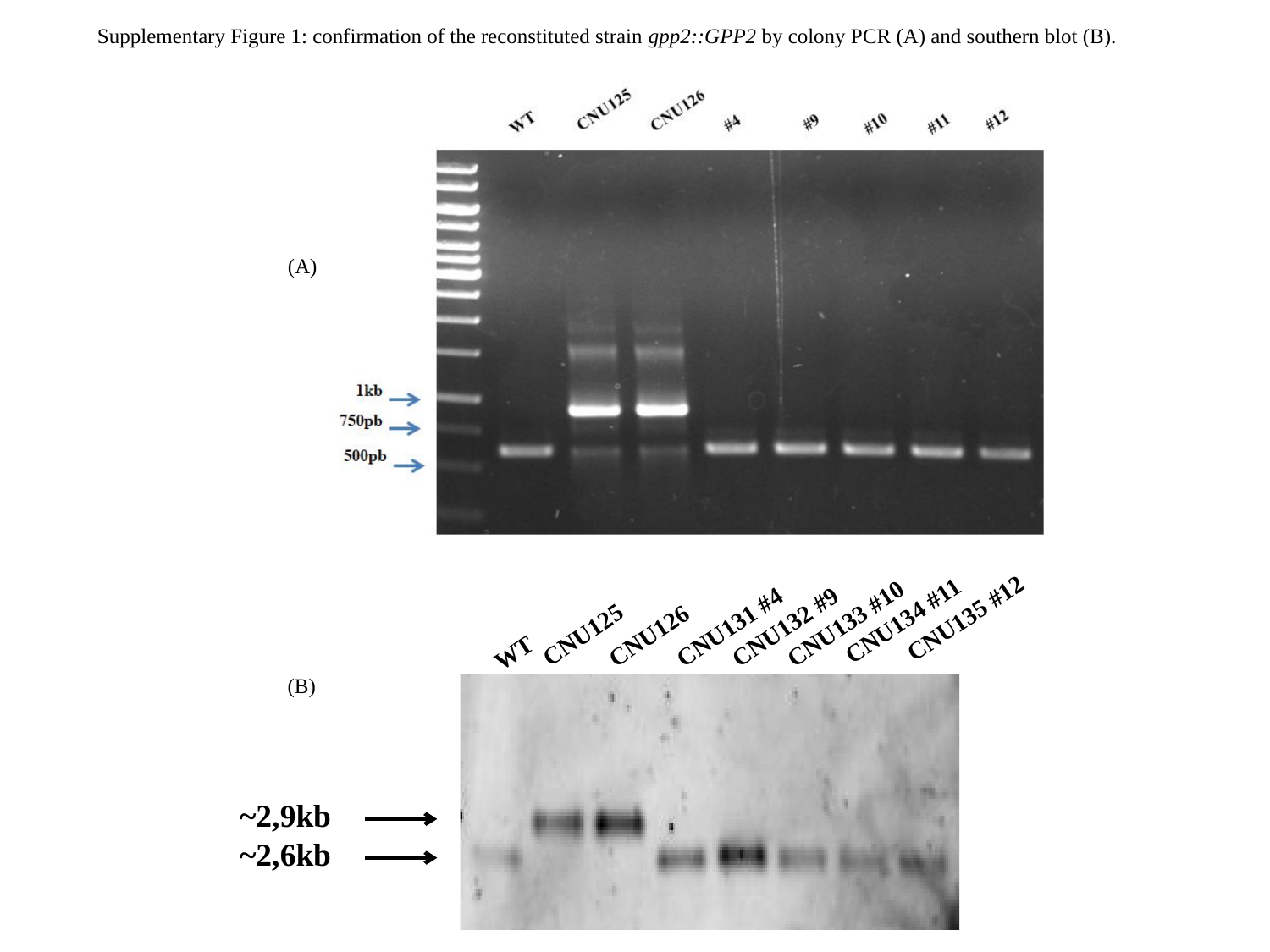

Supplementary Figure 1: confirmation of the reconstituted strain gpp2::GPP2 by colony PCR (A) and southern blot (B).
(A)
CNU135 #12
CNU134 #11
CNU131 #4
CNU133 #10
CNU132 #9
CNU125
CNU126
WT
~2,9kb
~2,6kb
(B)

## Slide 2
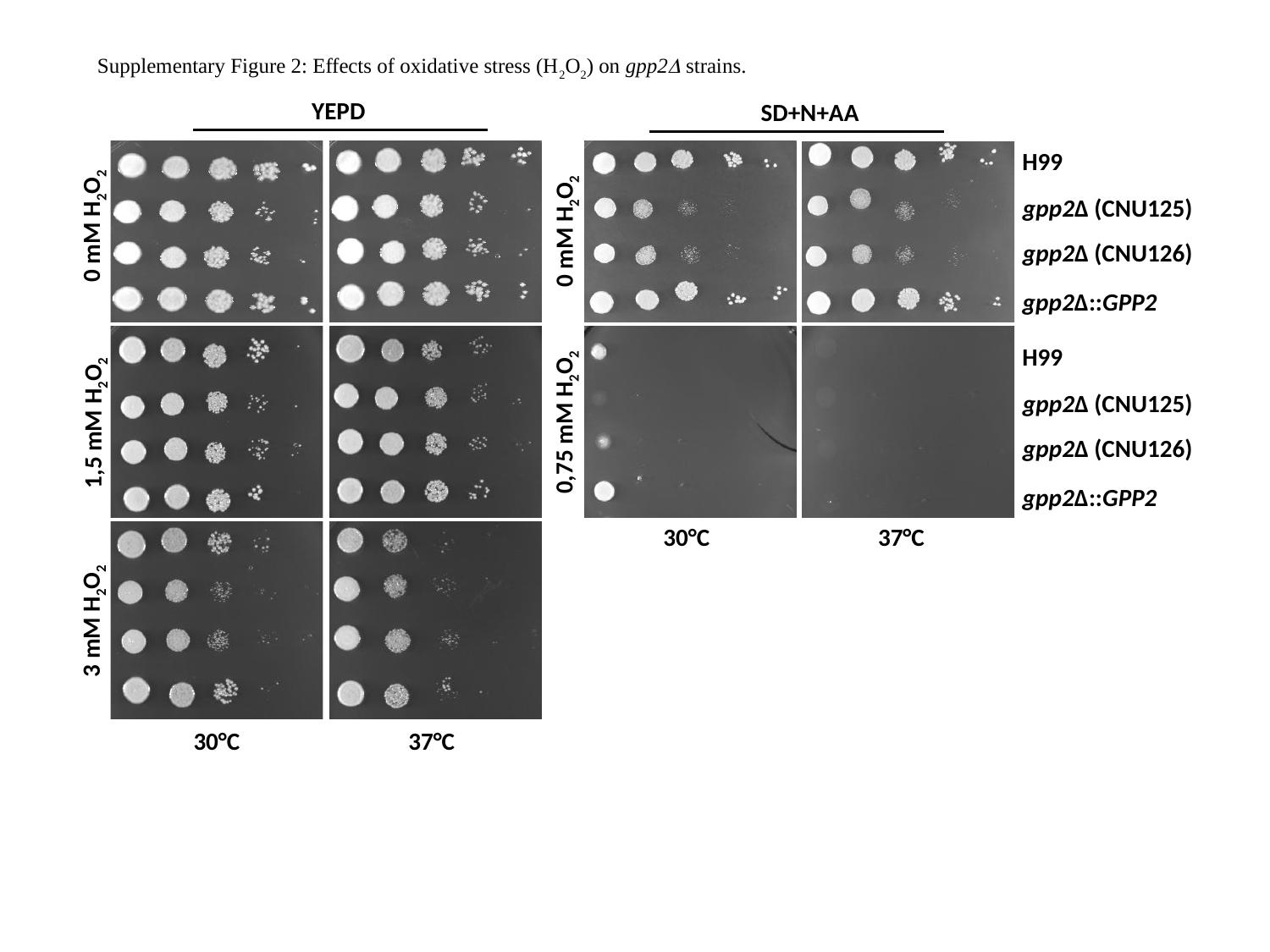

Supplementary Figure 2: Effects of oxidative stress (H2O2) on gpp2Δ strains.
YEPD
SD+N+AA
H99
gpp2Δ (CNU125)
 0 mM H2O2
0 mM H2O2
gpp2Δ (CNU126)
gpp2Δ::GPP2
H99
gpp2Δ (CNU125)
0,75 mM H2O2
1,5 mM H2O2
gpp2Δ (CNU126)
gpp2Δ::GPP2
30°C
37°C
3 mM H2O2
30°C
37°C
